# Laminar distribution and arbor density of two functional classes of thalamic inputs to primary visual cortex

**DOI:** 10.1016/j.celrep.2021.109826

**Published:** 2021-10-12

**Authors:** Jun Zhuang, Yun Wang, Naveen D. Ouellette, Emily E. Turschak, Rylan S. Larsen, Kevin T. Takasaki, Tanya L. Daigle, Bosiljka Tasic, Jack Waters, Hongkui Zeng, R. Clay Reid

**Affiliations:** 1 Allen Institute for Brain Science, Seattle, WA 98109, USA; 2 Lead contact

## Abstract

Motion/direction-sensitive and location-sensitive neurons are the two major functional types in mouse visual thalamus that project to the primary visual cortex (V1). It is under debate whether motion/direction-sensitive inputs preferentially target the superficial layers in V1, as opposed to the location-sensitive inputs, which preferentially target the middle layers. Here, by using calcium imaging to measure the activity of motion/direction-sensitive and location-sensitive axons in V1, we find evidence against these cell-type-specific laminar biases at the population level. Furthermore, using an approach to reconstruct axon arbors with identified *in vivo* response types, we show that, at the single-axon level, the motion/direction-sensitive axons project more densely to the middle layers than the location-sensitive axons. Overall, our results demonstrate that motion/direction-sensitive thalamic neurons project extensively to the middle layers of V1 at both the population and single-cell levels, providing further insight into the organization of thalamocortical projection in the mouse visual system.

## In brief

Zhuang et al., investigate the functionally specific thalamocortical projection patterns in mouse primary visual cortex at both the population and single-axon levels. They find that the motion/direction-sensitive axons project extensively to the middle layers of primary visual cortex, challenging an existing hypothesis proposing a superficial targeting bias of these axons.

## Graphical Abstract

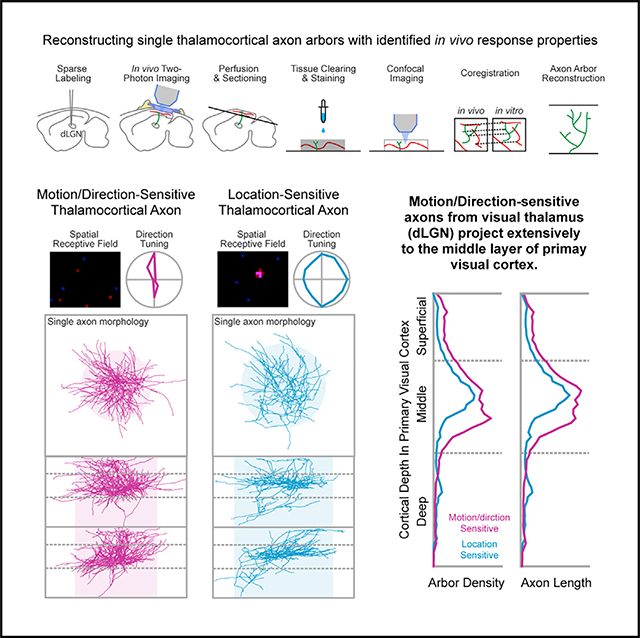

## INTRODUCTION

In mammalian visual systems, functionally specific thalamocortical projections from the dorsal lateral geniculate nucleus (dLGN) to the primary visual cortex (V1) serve as the major feedforward inputs for cortical computation. When compared with higher mammals, the mouse dLGN shows more diverse response properties. Particularly, besides cells with classical spatial receptive fields (RFs) that are sensitive to stimulus location (Grubb and Thompson, 2003; Denman and Contreras, 2016), a significant portion of the cells in mouse dLGN are sensitive to motion direction (Marshel et al., 2012; Piscopo et al., 2013; Zhao et al., 2013; Durand et al., 2016; Suresh et al., 2016; Román Rosón et al., 2019). How these motion/direction-sensitive dLGN cells project to V1 is not fully understood. One current view is that the motion/direction-sensitive cells, resembling the W cells in cats, preferentially project to the superficial layers (layer 1), in contrast to the location-sensitive (LS) cells, which have a middle layer bias (deep layer 3 and layer 4), resembling the X/Y cells in cats (Cruz-Martín et al., 2014; for a review, see Seabrook et al., 2017). However, the evidence for this laminar specificity is scarce and controversial: Whereas one study found, in support of this view, that the middle layers in V1 receive slightly less direction-sensitive inputs from the dLGN than the superficial layers (Kondo and Ohki, 2016), another study found contradictory evidence that middle and superficial layers receive similar amounts of direction-sensitive inputs from the dLGN (Sun et al., 2016). Furthermore, a recent large-scale survey of single-cell whole brain morphology did not find dLGN cells that preferentially target superficial layers in V1 (Peng et al., 2020). To address this inconsistency, we investigated the projection patterns of motion/direction-sensitive and LS dLGN axons in V1 at the population level and, more powerfully, at the single-axon level. We found strong evidence that the motion/direction-sensitive dLGN axons project extensively to the middle layers in V1, providing an alternative model of functional specificity in mouse thalamocortical projections.

## RESULTS

**Figure 1.**
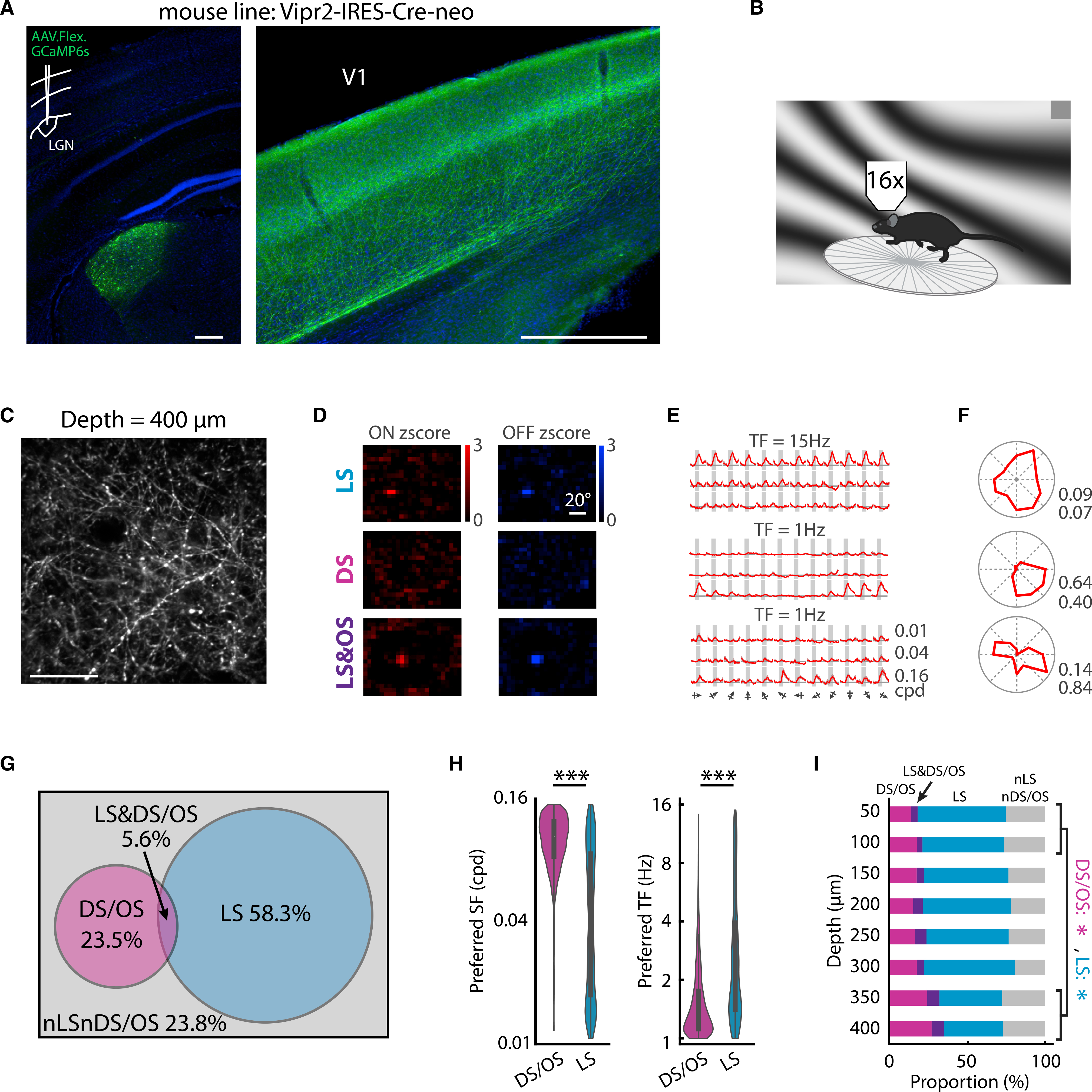
Labeling strategy, calcium imaging, classification, and depth distribution of dLGN boutons in V1 (A) GCaMP expressions in a mouse prepared for calcium imaging experiments. Coronal section. Inset: sketch for viral injection. Blue indicates DAPI; green indicates GCaMP6s. Scale bars, 500 μm. (B) Sketch of *in vivo* imaging setup. (C) Mean projection of two-photon calcium images from an example imaging plane at 400 μm below pia. Scale bar, 50 μm. (D–F) The ON and OFF *Z*-score RFs, averaged calcium responses to gratings, and direction tuning curves, respectively, of three example putative boutons. Traces in (E): mean df/f ± standard deviation across trials. Only responses to peak temporal frequency are indicated. For each ROI, responses were normalized to peak responses. Gray boxes: time windows used to calculate mean response amplitudes. Columns: direction. Rows: spatial frequency. LS: location sensitive. DS: direction sensitive. OS: orientation sensitive. Numbers in (F): top, gDSI; bottom, gOSI. Scale bar, 20 visual degrees. (G) Venn diagram describing the relations among the four different functional groups. (H) Distributions of preferred spatial frequencies and temporal frequencies of DS/OS and LS boutons. 4,694 DS/OS boutons versus 11,646 LS boutons; ***p < 0.001, Wilcoxon rank-sum test. Bar graph represents mean ± SEM. (I) The proportion of each of the four groups in (G) across cortical depths. The proportion of DS/OS boutons at deep depths (350, 400 μm) were higher than at superficial depths (50, 100 μm), and the LS boutons had an opposite trend. *p < 0.05.

To measure the population distribution of different dLGN inputs to V1, we labeled dLGN cells and their axons with calcium indicator by injecting adeno-associated viruses (AAVs) containing Cre-dependent GCaMP6s into the dLGN of Vipr2 (vasoactive intestinal peptide receptor 2)-IRES2-Cre-neo transgenic mice (Figure 1A), which have concentrated Cre expression in the dLGN (Figure S1). We then measured the calcium activity of labeled dLGN axons in V1 in awake, head-fixed animals using two-photon imaging (Figures 1B and 1C). We mapped the spatial RFs with locally sparse noise and measured the tuning for orientation/direction and spatial/temporal frequency (SF/TF) with full-field drifting gratings. A subset of experiments was carried out using adaptive optics to increase the fluorescence intensity and signal-to-noise ratio (Figure S2). The response properties were similar with and without adaptive optics (Figure S2), so the data were pooled together.

In total, 114 planes from 6 mice were imaged, with imaging depths ranging from 50 to 400 μm below the pia, from layer 1 through layer 4. Calcium traces were extracted from 40,008 regions of interest (ROIs) representing putative axonal boutons, among which 19,967 were responsive to at least one type of displayed visual stimulus (Table S1; STAR Methods). Within these responsive boutons, many showed significant spatial RFs (RF strength ≥ 1.6; Figure 1D; STAR Methods) indicating sensitivity to stimulus locations (LS boutons), and many showed strong selectivity to one grating direction (global direction selectivity index [gDSI] > 0.5; direction sensitive [DS]; Figures 1E and 1F, middle) or to two opposite grating directions (global orientation selectivity index [gOSI] > 0.5; orientation-sensitive [OS] boutons; Figures 1E and 1F, bottom) indicating sensitivity to visual motion directions. The LS group (11,646 boutons; 58.3%) and the DS/OS group (4,694 boutons; 23.5%) were largely separate, with only 5.6% (1,125 boutons) overlap (Figure 1G; Table S1), which was far less than chance (0.583 × 0.235 = 13.7%, p = 0.000, chi-square test). Consistent with previous electrophysiological studies (Piscopo et al., 2013), the DS/OS boutons showed a distinct preference to higher SF and lower TF compared to the LS boutons, which showed a broader range of SF and TF preference (Figure 1H; DS/OS versus LS; preferred SF: 0.10 ± 0.02 versus 0.04 ± 0.01 cycle per degree (cpd), p = 1.6 × 10^−14^; preferred TF: 1.50 ± 0.31 versus 2.68 ± 0.61 Hz, p = 3.3 × 10^−14^, Wilcoxon rank-sum test). A small number of boutons showed distinct types of response properties such as suppressed-by-contrast (data not shown), as reported previously (Piscopo et al., 2013; Durand et al., 2016; Suresh et al., 2016). Since they were relatively rare and were not the focus of this study, they were assigned to the non-LS and non-DS/OS (nLSnDS/OS) group. Consistent with one previous report (Sun et al., 2016), both LS and DS/OS boutons were distributed across all measured cortical depths, from 50 to 400 μm. The only notable bias was that relatively more DS/OS than LS boutons were found at the greatest depths compared to the most superficial depths (Figure 1I; Table S1; 50–100 μm versus 350–400 μm; proportion of DS/OS boutons: 15.6 ± 2.1% versus 23.3 ± 1.7%, p = 0.011; proportion of LS boutons: 61.4 ± 3.7% versus 47.5 ± 3.3%, p = 0.028, Mann-Whitney U test), providing evidence against the hypothesis that DS/OS axons target superficial layers preferentially (Cruz-Martín et al., 2014; Seabrook et al., 2017). In addition, all measured response metrics showed no depth bias except for preferred SF, which was higher at greater depths (Figure S3). Thus, LS and DS/OS boutons appear to form two distinct feedforward input types in V1 that are present in both superficial (layer 1 and superficial layer 2/3) and middle (deep layer 2/3 and layer 4) layers.

**Figure 2.**
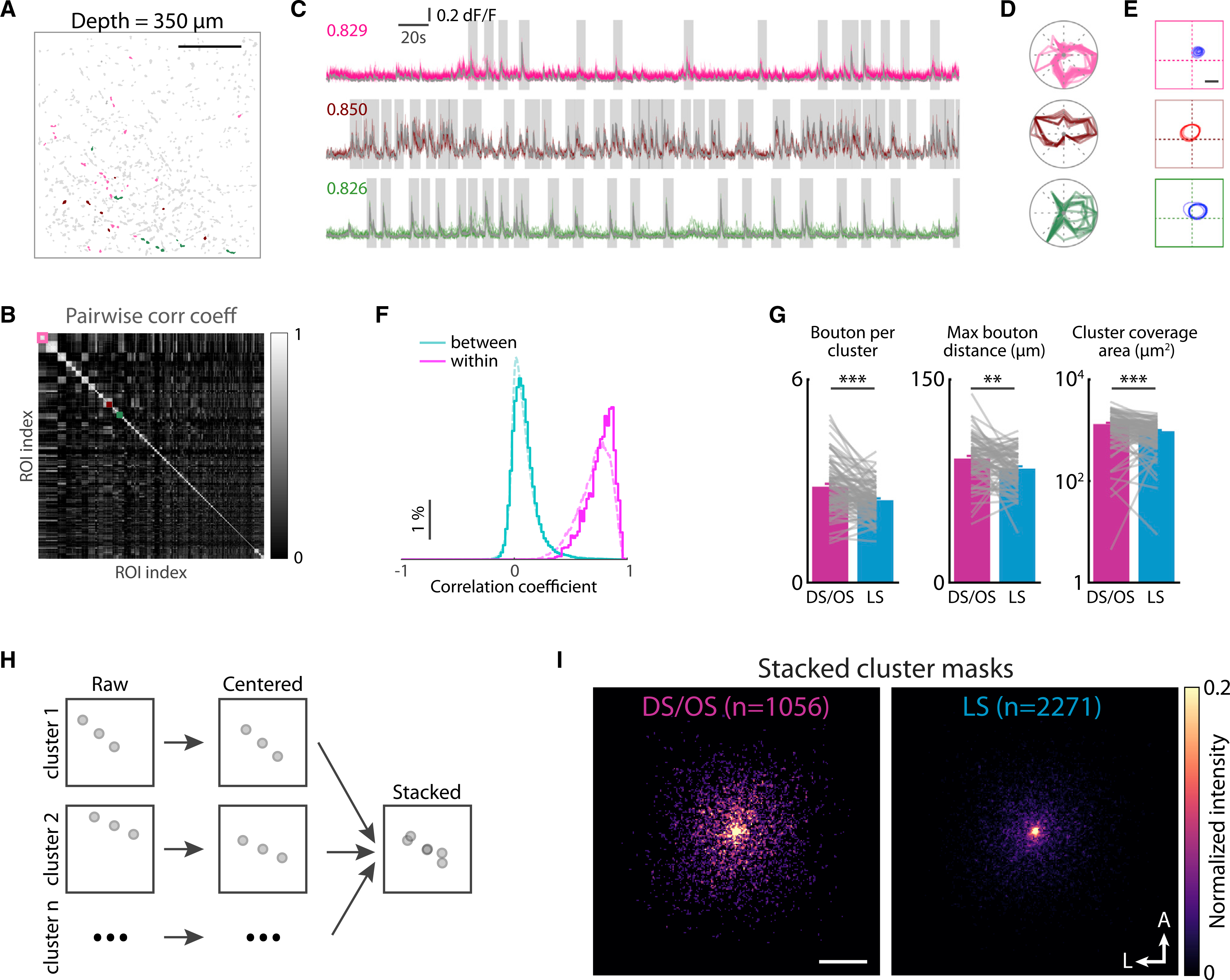
DS/OS bouton clusters have larger bouton spread than LS bouton clusters (A) An example imaging plane showing all active boutons (gray) and three example bouton clusters (colored). Scale bar, 50 μm. (B) Clustered correlation coefficient matrix for the imaging plane in (A). Example clusters in (A) are indicated by colored boxes. (C) Calcium traces from the example clusters in (A). Each row represents a cluster with matching color. Colored traces indicate traces from each bouton in the cluster. Gray traces indicate weighted average traces for each cluster. Numbers: mean pairwise correlation coefficients. Gray boxes indicate time windows of detected calcium events used for performing correlation (see STAR Methods). (D and E) Superimposed direction tuning curves and RF contours, respectively, of individual boutons from each cluster. Scale bar, 10 degrees. (F) Normalized distribution of correlation coefficients between within-cluster boutons and cross-cluster boutons. Solid line indicates data from the example plane in (A). Dashed line indicates data from all imaging planes. (G) Comparisons of bouton per cluster, maximum bouton distance, and cluster coverage area between DS/OS and LS clusters. Means of each matric were calculated for each plane and paired comparisons were made between DS/OS and LS clusters. Each gray line represents one imaging plane (n = 78 planes). **p < 0.01; ***p < 0.001, Wilcoxon rank-sum test, mean ± SEM. (H) Stacked masks were generated by superimposing centered individual cluster masks. (I) Stacked masks showing DS/OS clusters having larger bouton spread than LS clusters. A, anterior; L, lateral. Scale bar, 50 μm.

In analyzing a population, it is important to link together boutons that arise from the same axon, which we achieved by using a correlation-based hierarchical clustering procedure (Liang et al., 2018; STAR Methods). With this procedure, boutons with highly correlated activities were grouped into separate clusters (Figures 2A and 2B), and the correlation coefficients showed non-overlapping distributions of within-cluster and between-cluster bouton pairs (Figure 2F). The boutons from single clusters showed highly correlated calcium activity (Figure 2C) and near-identical response properties (Figures 2D and 2E), further confirming the high likelihood of their being from same axons. For each cluster, we used summed calcium traces from all boutons, weighted by ROI intensities, to calculate response properties and used the same criteria to group them into DS/OS clusters and LS clusters.

We then compared the structural properties between the DS/OS and LS clusters. For each cluster, we measured the bouton number, maximum bouton distance, and cortical coverage area. For all three metrics, the values from DS/OS clusters were greater than those from LS clusters (Figure 2G; for DS/OS versus LS, 87 imaging planes, bouton number: 2.84 ± 0.08 versus 2.44 ± 0.05, p = 7.2 × 10^−6^; maximum bouton distance: 91.5 ± 2.0 μm versus 84.3 ± 1.6 μm, p = 0.002; axon coverage: 1,324 ± 81 μm^2^ versus 957 ± 51 μm^2^, p = 3.8 × 10^−6^, Wilcoxon rank-sum test). To directly visualize the bouton spread of DS/OS and LS clusters, we generated stacked population cluster masks for each group (Figure 2H; STAR Methods). The results showed that the DS/OS clusters had substantially greater bouton lateral spread than the LS clusters, consistent with aforementioned measurements (Figure 2I; distances from every bouton to its cluster center, only clusters with more than one bouton were included, 1,056 DS/OS clusters versus 2,271 LS clusters, 42.2 ± 27.3 μm versus 39.7 ± 27.9 μm, p = 1.22 × 10^−8^, Mann-Whitney test). The greater bouton count and wider coverage of DS/OS cluster suggests that the DS/OS axons had denser axon arbors than the LS axons.

**Figure 3.**
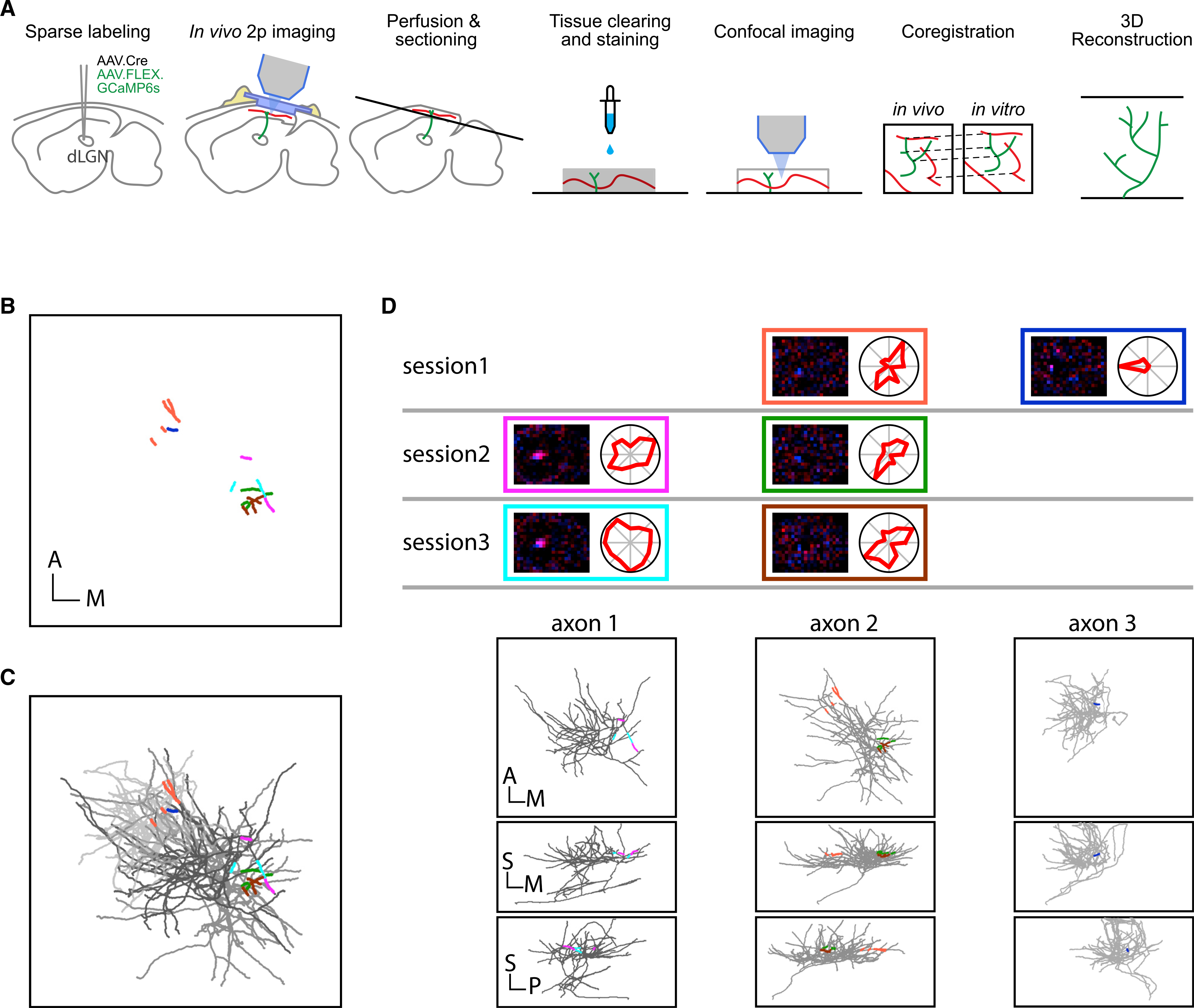
Reconstructing 3D structures of axons with identified *in vivo* response properties (A) Sketches indicating the workflow to reconstruct 3D structures of axons with identified *in vivo* response properties. dLGN axons were sparsely labeled by injecting amixture of AAV9-hSyn-Cre and AAV1-Syn-FLEX-GCaMP6s (1:40,000) into the dLGN of wild-type mice. Blood vessels were labeled by fluorescent dyes in both *in vivo* and *in vitro* imaging experiments and used as fiducials for coregistration across imaging modalities. Green lines indicate labeled axons. Red lines indicate labeled blood vessels. (B) Six axon segments recorded from a single mouse across three imaging sessions. Each color represents a bouton cluster identified using the calcium activity correlation-based clustering method (Figure 2). In total, 6 axon segments were identified. Scale bar, 100 μm. (C) Three reconstructed axon arbors (indicated as different gray levels) containing the 6 segments marked in (B). (D) Top: the RFs and direction tuning curves from the 6 segments indicated in (B) with color-matched boxes. Note that the segments imaged in different sessions can show similar (within-column) or different (cross-column) response properties. Bottom: three views of individual axon arbors in (C) showing that the segments having similar response properties across different imaging sessions belong to same arbors and that the segments having different response properties belong to different arbors. A, anterior; P, posterior; M, media; S, superficial. Scale bar, 100 μm.

**Figure 4.**
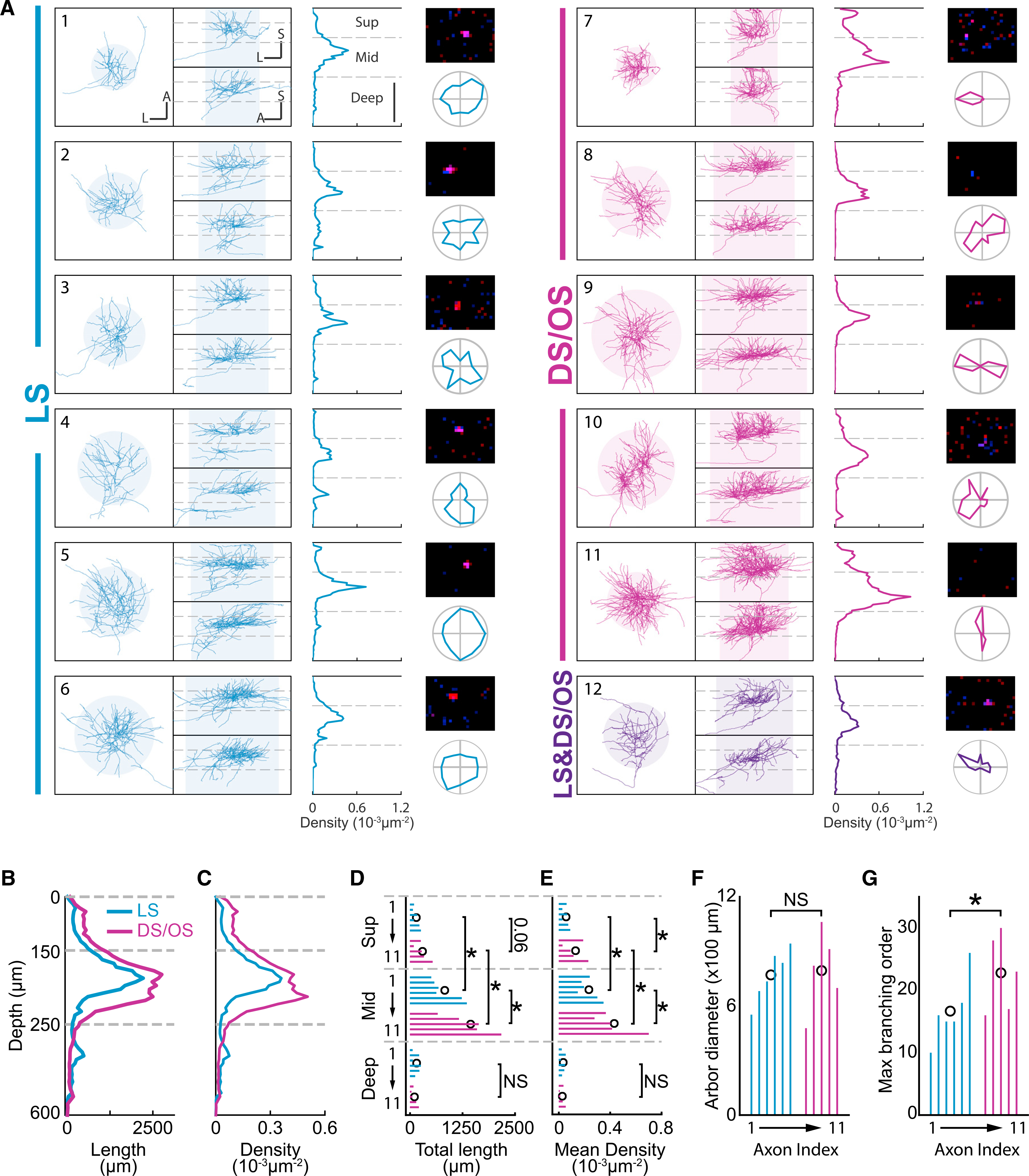
Single DS/OS axons project more extensively to superficial and middle layers than LS axons (A) 3D structures and response properties of all reconstructed axons. For each axon, the three views (left), axon density across depth (middle), RF (top right), and direction tuning curve (bottom right) are shown. Scale bar, 200 μm. A, anterior; L, lateral; S, superficial. Horizontal dashed lines indicate borders to define layers as superficial (0–150 μm), middle (150–250 μm), and deep (250–600 μm). Shaded circles indicate horizontal arbor extent defined as a circle extending from the center of mass and encompassing 95% of all segments. Shaded boxes represent volumes by extruding the arbor extents along cortical depth for calculating arbor density. Numbers indicate indices for each axon that match those in (D)–(G). (B and C) Comparisons of mean total length and mean density, respectively, across cortical depth between DS/OS and LS axons. Horizontal dashed lines match those in (A). (D and E) Comparisons of total length and density, respectively, between the DS/OS and LS axons in superficial, middle, and deep layers (separated by horizontal dashed lines) in (A). Colored bars represent measurements from single axons ordered by the indices in (A). Open black circle indicates the mean for each group at each depth. *p < 0.05. NS, not significant (p > 0.05), Mann-Whitney test. (F and G) Comparisons of arbor diameter and maximum branching order, respectively, between the DS/OS and LS axons in (A). Colored bars represent measurements from single axons ordered by the indices in (A). Open black circle indicates the mean. *p < 0.05. NS, not significant (p > 0.05), Mann-Whitney test.

Using *in vivo* two-photon images to estimate axon structure, however, led to highly incomplete sampling; a two-photon imaging plane (~180 × 180 × 6 μm) only sampled less than 1% of a typical axon arbor, which can extend to a volume of ~500 × 500 × 500 μm (Antonini et al., 1999; Figure 4). To overcome this limitation, we developed an approach that allowed us to investigate the structure of complete axon arbors with identified *in vivo* response properties (Figure 3A; STAR Methods). In this approach, dLGN axons were sparsely labeled with GCaMP6s, and blood vessels were labeled with a red fluorescent dye (Dextran Texas Red). The same aforementioned visual stimuli and imaging protocols were used to identify response types. The brain tissue was then fixed and sectioned tangentially into thick (350–400 μm) sections, followed by antibody staining to enhance GCaMP signal and a counterstain to label blood vessels. Finally, the tissue was cleared using the CUBIC clearing method (Susaki et al., 2015). The labeled and cleared tissue volumes were then imaged with a confocal microscope. Using the labeled blood vessels, the *in vivo* two-photon images were spatially aligned to the confocal image stacks (Figure S4), allowing precise coregistration between the *in vivo* functional recordings of the boutons and their anatomical locations followed by tracing and 3D reconstruction. Due to the high sparsity of the labeling, different boutons with very similar response properties (even across different imaging sessions) always appeared to belong to same reconstructed axons (Figures 3B–3D), confirming the reliability of our coregistration and the completeness of our reconstructions.

In total, 12 axons—six LS, five DS/OS, and one LS&DS/OS—with robust responses were successfully reconstructed (Figure 4A). All axons projected extensively to the middle layers (150–250 μm below pia) and extended to the superficial layers (0–150 μm below pia). Three LS axons (axons 2, 4, and 5 in Figure 4A) but no DS/OS axons showed secondary clusters in the deep layers (250–600 μm below pia). Although, surprisingly, no significant differences were found in the horizontal coverage (shaded circles in Figure 4A; STAR Methods) between LS and DS/OS axons (Figure 4F; coverage diameter, DS/OS versus LS, 794 ± 221 μm versus 770 ± 142 μm, p = 0.46, Wilcoxon rank-sum test), there were prominent differences in segment length and arbor density (defined as segment length divided by a cylindrical volume encompassing the axon; STAR Methods) between superficial and middle layers and between DS/OS and LS axons. First, both DS/OS and LS axons showed greater segment length and arbor density in the middle layers than in the superficial layers (Figures 4B–4E; middle versus superficial, DS/OS length: 28.9 ± 11.3 mm versus 5.9 ± 3.2 mm, p = 0.03; LS length: 16.5 ± 7.3 mm versus 3.0 ± 1.6 mm, p = 0.02; DS/OS density: 43.1 ± 15.9 μm^−2^ versus 13.0 ± 8.0 × 10^−5^ μm^−2^, p = 0.03; LS density: 23.4 ± 7.7 μm^−2^ versus 5.7 ± 2.5 × 10^−5^ μm^−2^, p = 0.02, Wilcoxon rank-sum test). In addition, when compared with the LS axons, the DS/OS axons had greater segment length and arbor density in the middle layers (Figures 4B–4E; length: p = 0.04; density: p = 0.01, Mann-Whitney test). This difference was also apparent in the superficial layers, but only the difference in arbor density reached statistical significance (length: p = 0.06; density: 0.03, Mann-Whitney test). No significant difference was found in the deep layers (length: p = 0.31; density: p = 0.51, Mann-Whitney test). Finally, the DS/OS arbors showed higher maximum branching order than the LS axons consistent with their higher arbor density (Figure 4G; DS/OS versus LS, 22.8 ± 6.3 versus 16.7 ± 5.3, p = 0.049, Mann-Whitney test). Overall, the single-axon reconstruction results indicated that DS/OS axons, like the LS axons, preferentially target the middle layers, but with denser and more complex axon arbors. This is consistent with our population data showing the distribution of DS/OS boutons biased toward middle layers (Figure 1I; Table S1).

## DISCUSSION

In this study, we show strong evidence, at both the population and single-cell levels, that the motion/direction-sensitive dLGN neurons project extensively to the middle layers in V1, arguing against the hypothesis predicting their superficial projection bias (Seabrook et al., 2017) but consistent with the findings from other species (Hei et al., 2014; Bereshpolova et al., 2019). These results suggest that the motion sensitivity in the V1 middle-layer cells can be partially inherited from the dLGN motion/direction-sensitive inputs, although it can also be constructed from non-direction-sensitive dLGN inputs (Lien and Scanziani, 2018). Our results also argue against the proposed homology between the DS/OS pathway in mice and the W pathway in cats (Seabrook et al., 2017). The DS/OS cells in this study project heavily to middle layers (Figure 4) and preferred lower TF and higher SF than LS cells did (Figure 1H, Piscopo et al., 2013), whereas the W cells in cat dLGN predominantly project to the superficial layers (Anderson et al., 2009) and prefer lower SF than X and Y cells did (Sur and Sherman, 1982). Interestingly, our data show that the boutons in middle layers have higher peak SF than boutons in superficial layers (Figure S3E), which is consistent with the depth dependency of peak SF in mouse cortical neurons (de Vries et al., 2020). Given that, in other mammals, the dLGN afferents preferring higher SF (X afferents in cats, parvocellular afferents in monkeys) project to deeper layers than the afferents preferring lower SF (Y afferents in cats, magnocellular afferents in monkeys; Gilbert and Wiesel, 1979; Humphrey et al., 1985; Jin et al., 2008; for a review, see Nassi and Callaway, 2009), this depth-dependent SF preference may indicate an important organization of mammalian thalamocortical system.

Neurons in this study were targeted with local viral injections combined with the newly developed Vipr2-IRES2-Cre-neo transgenic mice to label dLGN projecting neurons (Figure 1A). Although a small number of Cre+ cells can be found in higher order visual thalamus (lateral posterior nucleus; LP) in this mouse line (Figure S1), local injection into dLGN minimized the Cre dependent GCaMP expression in this region (Figure 1A). In dLGN, the Cre+ cells account for ~35%–50% of all Nissl-stained cells (Figure S1B). Given that Nissl stain labels both neuronal and non-neuronal cells, the ratio of Cre+ cells to dLGN neurons will be significantly higher. Additionally, the gOSI distributions (Figure S3C) of our data are similar to those reported by a previous imaging study using wild-type and other transgenic mice (Sun et al., 2016), and the LS-DS/OS dichotomy found in our data (Figures 1G and 1H) is qualitatively similar to that found in previous electrophysiology studies (Piscopo et al., 2013; Román Rosón et al., 2019), suggesting that the Cre+ cells in Vipr2-IRES2-Cre-neo mice functionally represent the overall dLGN neuron population.

Compared to the rapid progress in mapping complete local functional connectomes in mouse V1 (https://www.microns-explorer.org), our knowledge about how V1 receives its major inputs from dLGN is surprisingly incomplete. A recent large-scale survey provided morphological classifications of thalamocortical projections (Peng et al., 2020), but without *in vivo* physiology, the insights it can provide for cortical computation are indirect. Our study, with its unique strength in linking *in vivo* functions and single-axon morphologies, complements these large-scale studies by providing function-structure correspondence of the major feedforward inputs to V1.

## STAR⋆METHODS

### RESOURCE AVAILABILITY

#### Lead contact

Further information and requests for resources and reagents should be directed to and will be fulfilled by the Lead Contact, Jun Zhuang (junz@alleninstitute.org).

#### Materials availability

The DNA plasmids generated in this study (AAV pCAG-mRuby3-WPRE) can be acquired from Addgene (Catalog#: 107744). The Vipr2-IRES2-Cre-neo mouse line is in the process of being deposited to Jackson Laboratory at the time of this writing.

#### Data and code availability

All custom code used in this study is available in the github repositories “NeuroAnalysisTools” (https://doi.org/10.5281/zenodo.5512966), and “retinotopic_mapping” (https://doi.org/10.5281/zenodo.5512971). The data that support the findings of this study are available from the lead contact, Jun Zhuang (junz@alleninstitute.org), upon reasonable request. Any additional information required to reanalyze the data reported in this paper is available from the lead contact upon request.

### EXPERIMENTAL MODEL AND SUBJECT DETAILS

Both male and female transgenic mice older than P56 were utilized for all experiments (injection and cranial window surgeries were done between P61 and P72, *in vivo* imaging experiments were done between P103 and P232). All animals were housed 3–5 per cage before surgery and single housed after surgery and maintained on a 12-hour light/dark cycle. All experimental procedures related to the use of mice were conducted with approved protocols in accordance with NIH guidelines, and were approved by the Institutional Animal Care and Use Committee of the Allen Institute for Brain Science.

### METHOD DETAILS

#### Surgery and animal preparation

For the dense labeling experiments, a 1:3 mixture of AAV1-CAG-mRuby3 (custom made from plasmid Addgene 107744, titer: 1.6×10^12^ vg/ml) and AAV1-Syn (or CAG)-FLEX-GCaMP6s (Addgene: 100845-AAV1, titer 4.3×10^13^ vg/ml or 100842-AAV1, titer 1.8×10^13^ vg/ml, respectively) was injected into the dLGN of six Vipr2-IRES2-Cre-neo mice (3 male, 3 female, 200nL each). For the sparse labeling experiments, a 1:1 mixture of AAV9-hSyn-Cre (1:40000 dilution, Addgene: 105553-AAV9, titer: 3.3×10^13^vg/ml) and AAV1-Syn-FLEX-GCaMP6s (Addgene: 100845-AAV1, title: 2.5×10^13^vg/ml) was delivered into the dLGN of 6 wild-type C57BL/6J mice (3 male, 3 female, 100nL each; Economo et al., 2016). Briefly, the injection pipette was slowly lowered into left dLGN (2.3 mm posterior, 2.3 mm lateral from bregma, 2.6 mm below pia) through a burr hole on the skull. Five minutes after reaching the targeted location, the virus mixture was injected into the brain over a period of 10 minutes using a hydraulic nanoliter injection system (Nanoject III, Drummond). The pipette then remained in place for an additional 10 minutes before it was slowly retracted out of the brain. Immediately after injection, a titanium head-plate and a 5 mm glass cranial window were installed over left V1 (Goldey et al., 2014) allowing for *in vivo* two-photon imaging during head fixation.

After surgery, the animals recovered for a minimum of 5 days before undergoing retinotopic mapping with intrinsic signal while anesthetized (Juavinett et al., 2017). After retinotopic mapping, animals were handled and habituated to the imaging rig for two additional weeks (de Vries et al., 2020) before *in vivo* two-photon imaging.

#### *In vivo* two-photon imaging

In awake animals, the calcium activities were recorded either with a conventional two-photon microscope or with a multi-plane two-photon microscope (described below). In both microscopes, a 16x/0.8 NA water immersion objective (Nikon 16XLWD-PF) was rotated to 24 degrees from horizontal to image visual cortex using a commercial rotating head (Sutter MOM). Emitted light was first split by a 735 nm dichroic mirror (FF735-DiO1, Semrock). The short-wavelength light was filtered by a 750 nm short-pass filter (FESH0750, Thorlabs) and a 470–588 nm bandpass emission filter (FF01–514/44–25, Semrock) before being collected as GCaMP signal, while the long-wavelength light was filtered by a 538–722 nm band-pass emission filter (FF01–630/92–30, Semrock) before being collected (mRuby for dense labeling experiments and Dextran-Texas Red for sparse labeling experiments). Image acquisition was controlled using Vidrio ScanImage software for both scopes (Pologruto et al., 2003; Vidrio LLC). To maintain constant immersion of the objective, we used gel immersion (Genteal Gel, Alcon) instead of water.

For experiments with dense labeling (6 mice), thalamocortical axons in V1 were imaged at 8 cortical depths (50, 100, 150, 200, 250, 300, 350, and 400 μm below pia). Imaging was done in a columnar fashion: at each cortical location calcium activities at 3–8 depths were imaged plane-by-plane over multiple sessions.

For experiments with sparse labeling/axon reconstruction (6 mice), an additional 30–60 uL of Dextran Texas Red (Thermo Fisher, D3328, 25 mg/mL solution with saline) was injected subcutaneously ~20 minutes before single-plane two-photon imaging sessions to label blood vessels. For each imaging session, a local z stack (field of view 358 × 358 μm from pia to depth of 500 μm with 4 μm step) was recorded to aid coregistration.

#### Single-plane two-photon imaging

Two-photon excitation was generated by laser illumination from a Ti:sapphire laser (Coherent Chameleon Ultra II) tuned to 920 nm. A single z-plane (179.2 × 179.2 μm) was imaged for during each session at a frame rate of about 30 Hz, with an 8 KHz resonate scanner (Cambridge Technology, CRS 8K). To maintain a constant imaging depth, automatic z-drift correction functions were implemented for experiments using the MOM motors. Prior to each imaging session, a correction z stack (±50 μm from targeted depth, 2 μm step depth) was acquired. During the session, the target imaging plane was continuously compared to each plane in the correction z stack. If a drift in depth was detected, the stage was automatically adjusted to compensate for the drift, thus maintaining a constant imaging depth. We found this procedure crucial to our experiments as boutons are small objects and a few-micron-drift in depth would result in imaging a different set of boutons.

#### Multi-plane two-photon imaging

In this custom-built, multi-plane two-photon microscope (DeepScope, Liu et al., 2019), a liquid crystal spatial light modulator (SLM; HSP-512, Meadowlark Optics) shapes the pupil wavefront to implement fast-focusing and adaptive optics. The objective pupil was slightly underfilled (~0.65 effective NA) and correction of systemic aberrations was performed with fluorescent beads to maintain near diffraction-limited focusing over a 200 um range. Two-photon excitation was produced by laser light from a commercial solid-state laser (Spectra-Physic Insight X3 laser) tuned to 940 nm. With this microscope, we simultaneously recorded calcium activity from planes at 5 different depths (50, 100, 150, 200, 250 μm) in single imaging sessions. Individual frames (125 × 125 μm with 512 × 512 pixels resolution) were acquired at an overall frame rate of ~37 Hz with a volume rate of 7.4 Hz. The DeepScope showed nearly zero z-drift for a prolonged duration (< 2 μm over 24 hours), so we did not implement z-correction during sessions using DeepScope.

In another set of experiments, we used DeepScope to assess the effect of adaptive optics adjusted on individual animals. The correction procedure was similar to the method described in Sun et al., 2016. For two mice, 1 μm beads (Thermo Fisher, F8821) were deposited on top of the brain surface under the coverglass during the initial surgery. Prior to the imaging session, modal optimization over 12 Zernike modes (up to j = 15 Noll ordering, excluding piston and tilt) was run to identify the SLM pattern that maximized the beads’ fluorescent signal. Then, during the imaging session, this SLM pattern was turned on and off alternatively for consecutive two-photon imaging frames (one frame on, one frame off, repeated) and drifting gratings were displayed. After imaging, the interleaving movie was separated into two movies: one with adaptive optics and the other without. Bouton tuning properties were then extracted from each movie and compared against each other.

All imaging sessions were performed during head fixation with the standard Allen Institute Brain Observatory *in vivo* imaging stage (de Vries et al., 2020).

#### Visual stimulation

All visual stimuli were generated and displayed by Retinotopic_Mapping python package (https://github.com/zhuangjun1981/retinotopic_mapping; Zhuang et al., 2017) over PsychoPy software (https://www.psychopy.org; Peirce et al., 2019) on a 24-inch LCD monitor (ASUS PA248Q, frame rate 60 Hz, 1920 × 1200 pixels, mean luminance 45.3 cd/m^2^) placed 15 cm from the mouse’s right eye (covering 120° × 95° of monocular visual space). We displayed locally sparse noise and full-field drifting grating in each imaging session to measure receptive fields and orientation/direction/spatial and temporal frequency tuning properties, respectively. In most sessions, we also displayed a five-minute full-field mid-luminance gray to measure spontaneous activity. For locally sparse noise, bright and dark squares (5° × 5°) were displayed in a random sequence on a grid tiling the entire monitor. At any given time, multiple squares could be displayed, but the minimum distance between those squares should be no less than 50°. Each square lasted 100 ms and in total was displayed 50 times. For drifting gratings, the combinations of 12 directions (every 30°), 3 spatial frequencies (0.01, 0.04 and 0.16 cpd), and 3 temporal frequencies (1, 4, 15 Hz) were displayed. Each display lasted 1 s and was spaced by 1 s mean luminance gray period. In total, 3 × 3 × 12 + 1 (blank) = 109 conditions were randomly displayed in each iteration and the entire sequence contained 13 iterations. Although the SFs and TFs presented in our stimulus set were not enough to map comprehensive tuning curves (limited by total imaging time of each session), they were sufficient to be used for comparisons between different functional groups. All stimuli were spherically corrected so that they were presented with accurate visual angles on the flat screen (Zhuang et al., 2017).

#### Two-photon image preprocessing

The recorded two-photon movies for each imaging plane were first temporally averaged across every 5 frames (Sutter scope) or 2 every frames (DeepScope) retaining an effective temporal frequency of ~6 Hz. Motion-correction was then performed on the red channel (mRuby in densely labeled samples and Texas Red in sparsely labeled samples) using a rigid body transformation based on phase correlation by a custom-written python package (https://github.com/zhuangjun1981/NeuroAnalysisTools; Zhuang et al., 2017). The resulting correction offsets were then applied to the green channel (GCaMP). To generate regions of interest (ROIs), the motion-corrected movies were further temporally downsampled by a factor of 3 and then processed with constrained non-negative matrix factorization (CNMF, Pnevmatikakis et al., 2016) implemented in the CaImAn python library (https://github.com/flatironinstitute/CaImAn; Giovannucci et al., 2019). The resulting ROIs were filtered by their size ([1.225, 12.25] μm^2^), position (ROIs within the motion artifacts were excluded) and overlap (for ROIs with more than 20% overlap, the smaller ones were excluded). For each retained ROI, a neuropil ROI was created as the region between two contours by dilating the ROI’s outer border by 1 and 8 pixels excluding the pixels within the union of all ROIs. The same procedures for neuropil subtraction used in our previous studies (Zhuang et al., 2017; de Vries et al., 2020) was then applied. As reported previously, a high skewness is an indication of active calcium activity (Mukamel et al., 2009; Dipoppa et al., 2018). Only the ROIs with skewness greater than 0.6 were defined as “active” boutons and were included in this study. For each imaging session, a comprehensive file in Neurodata Without Borders (nwb) 1.0 format was generated to store and share metadata, visual stimuli, all preprocessing results, and final calcium traces using the “ainwb” package (https://github.com/AllenInstitute/nwb-api).

#### Bouton clustering

The correlation-based bouton clustering procedure was based on previously reported algorithms (Petreanu et al., 2012; Liang et al., 2018) and the same procedure was performed on each imaging plane. First, for each ROI, calcium events were detected as up-crosses over a threshold of 3 standard deviations above the mean in its calcium trace (Gaussian filtered with a sigma of 0.1 s). A period of 3 s before and after each onset was defined as an event window and the union of all event windows for a particular ROI was saved. Second, for any given pair of boutons, the union of event windows from both boutons was generated and the calcium trace within the union window was extracted and concatenated for each bouton. A Pearson correlation coefficient of the two concatenated traces was then calculated for this pair. By performing this procedure on all pairs of active ROIs, we generated a correlation coefficient matrix for each imaging plane. We found the event detection to be important because it confined the correlation to the period in which at least one bouton in the pair was active, thus avoiding correlating the noise during the inactive period. Third, the correlation coefficient matrices were further thresholded to reduce noise: the correlation coefficients for a given bouton were maintained if the coefficients were larger than 0.5 or if they exceeded 3 standard deviations above the mean value of all the coefficients between this bouton and all others. Otherwise, they were set to 0. Third, a hierarchy clustering was performed to a given imaging plane using “1 – thresholded correlation coefficient matrix” as the distance matrix using Scipy.cluster.hierarchy library with a “weighted” method (https://docs.scipy.org/doc/scipy/reference/generated/scipy.cluster.hierarchy.linkage.html). Fourth, we use a threshold of 1.3 to separate clusters since ~1.5 shows up as a relatively natural cut-off in the dendrograms. This threshold appeared somewhat conservative on the clustered correlation coefficient matrix. Fifth, a calcium trace of each bouton cluster was calculated as a mean calcium trace of all boutons belonging to this cluster, weighted by the sum of their ROI masks.

For each bouton cluster, three values were extracted to estimate the morphology: (1) bouton number of this cluster; (2) maximum distance among all bouton pairs belonging to this cluster; (3) area of the convex polygon encapsulated by all the boutons belonging to this cluster. For axons with only one bouton, the metrics 2 and 3 were set to be “nan” and were excluded from statistical analysis.

To generate stacked cluster masks, we first selected bouton clusters with at least two boutons for each group. The binary mask of each individual cluster was then centered to its own center of mass. All centered masks were summed together to generate a summed population mask for each group, normalized by its peak. LS&DS/OS bouton clusters were excluded from this analysis to avoid double counting.

#### Spatial receptive field analysis

We calculated the units’ spatial receptive fields from their responses to the locally sparse noise stimulus using reverse correlation analysis (Zhuang et al., 2013, 2017). For each stimulus location, the df/f value was calculated as (response – baseline) / baseline (with mean calcium trace [0, 0.5] second after stimulus onset as response and [−0.5, 0] second before onset as baseline). From this df/f amplitude map, a z-score map was calculated by subtracting the mean and dividing the standard deviation of the entire map. The z-score map was then smoothed (Gaussian filter, sigma = 1 pixel) and up-sampled by a ratio of 10 with cubical interpolation. RF strength was defined as the peak value of this z-score map. ROIs with an RF strength no less than 1.6 were defined as having a significant spatial RF. For each significant spatial RF, an RF mask was generated by thresholding, either with a value of 1.6 (for maps with a peak less than 4) or with a value of 40% of its peak (for maps with a peak greater than 4).

#### Grating response analysis

The units’ responses to drifting gratings were analyzed by the event-triggered average procedure similar to the RF response analysis (0.5 s before onset as baseline and 1 s after as response). We applied the same inclusion criteria as in our previous large-scale study (de Vries et al., 2020). The direction tuning curve was extracted at peak TF/SF conditions and, if the minimum response from this curve was below zero, an offset was added to the whole curve so that the minimum was zero. The SF and TF tuning curves were extracted using a similar procedure.

We calculated global direction selectivity index (gDSI) as

$$gDSI=\frac{\left|\sum R_{j}e^{i\theta_{j}}\right|}{\sum R_{j}},$$

global orientation selectivity index (gOSI) as

$$gOSI=\frac{\left|\sum R_{j}e^{i2\theta_{j}}\right|}{\sum R_{j}},$$

and the preferred direction as the angle of $\sum R_{j}e^{i\theta_{j}}$. Where j represents different direction conditions, R represents df/f response in each direction and θ represents the direction. We defined a unit to be orientation-selective if its gOSI was greater than 0.5, and we defined a unit to be direction-selective if its gDSI was greater than 0.5.

From the SF tuning curve, we calculated the preferred SF as

$$peak\;SF=2^{\left(\frac{\sum R_{j}log_{2}SF_{j}}{\sum R_{j}}\right)}$$

Where j represents different SF conditions. From TF tuning curve, we calculated the preferred TF as

$$peak\;TF=2^{\left(\frac{\sum R_{j}log_{2}TF_{j}}{\sum R_{j}}\right)}$$

Where j represents different TF conditions.

#### Perfusion

For the histology experiments, the brain tissue was collected by transcardial perfusion. Mice were anesthetized with 5% isoflurane, then 10 mL of saline (0.9% NaCl) followed by 50 mL of freshly prepared 4% paraformaldehyde (PFA) were pumped intracardially at a flow rate of 9 ml/min. Brains were immediately dissected and post-fixed in 4% PFA at room temperature for 3–6 hours and then overnight at 4°C. After fixation, brains were incubated in PBS with 10% sucrose and then stored in PBS with 30% sucrose until sectioning.

#### Histology for expression characterization

To characterize the Cre expression pattern, Vipr2-IRES2-Cre-neo mice were crossed with the Ai14 reporter line (Madisen et al., 2010) to generate Vipr2-IRES2-Cre-neo/wt; Ai14/wt animals. The brains of these animals were cut into 50 μm sections using a freezing-sliding microtome (Leica SM 2101R). Sections with dLGN and V1 were then mounted on gelatin-coated slides and coverslipped with mounting media (Prolong Diamond Antifade Mounting Media, P36965, ThermoFisher).

To verify the injection location and GCaMP expression, the brain tissue from mice in dense labeling experiments was collected and sectioned using a similar procedure after all imaging sessions. Additional immunohistochemistry steps were performed to enhance the GCaMP signal before mounting and coverslipping. During antibody staining, sections containing dLGN and V1 were blocked with 5% normal donkey serum and 0.2% Triton X-100 in PBS for one hour, incubated in an anti-GFP primary antibody (1:5000 diluted in the blocking solution, Abcam, Ab13970) for 48–72 hours at 4°C, washed the following day in 0.2% Triton X-100 in PBS, and incubated in an Alexa 488 conjugated secondary antibody (1:500 diluted in the blocking solution, 703–545-155, Jackson ImmunoResearch) and DAPI.

The sections were then imaged with Zeiss AxioImager M2 widefield microscope with a 10x/0.3 NA objective. Fluorescence from antibody-enhanced GCaMP and mRuby3 were extracted from filter sets Semrock GFP-1828A (excitation 482/18 nm, emission 520/28 nm, dichroic cutoff 495 nm) and Zeiss # 20 (excitation 546/12 nm, emission 608/32 nm, dichroic cutoff 560 nm), respectively.

#### Histology for axon reconstruction

To reconstruct the sparsely labeled dLGN axons, brain sections from mice in the sparse labeling experiments were cut tangentially to the imaging window surface with thickness of 350 or 400 μm on a vibratome (Leica VT1000, modified from Zhuang et al., 2018). The sections were then (1) incubated in PBS with 8% SDS for 48 hours at 37°C for initial clearing; (2) blocked by blocking solution NDSTU (5% Normal Donkey Serum, 4M Urea, 0.2% Triton X-100) at room temperature for 1 hour; (3) incubated in primary antibody for GFP (1:5000 in NDSTU, Abcam, Ab13970) at room temperature for 48 hours; (4) incubated in secondary antibody (1:500 in NDSTU, 703–545-155, Jackson ImmunoResearch) and lectin (for labeling blood vessels, 2 μg/mL, Vector Laboratories, DL1178) at room temperature for 48 hours; and (5) mounted with CUBIC tissue clearing solution (Urea 25 wt%, Quadrol 25 wt%, Triton X-100 15 wt% in dH_2_O, Susaki et al., 2015) with spacers of appropriate depth (SunJin Lab, iSpacer).

The relevant regions (field of view 1.7 × 1.7 mm or 1.7 × 1.2 mm) were then imaged by a confocal microscope (Olympus FV3000) as tiled z stacks (30x oil immersion objective with resolution 0.414 (x) × 0.414 (y) × 0.5 (z) μm, excitation 488 nm / emission [500, 540] nm for axons and excitation 640 nm / emission [650, 750] nm for blood vessels).

#### Coregistration, reconstruction, and morphology analysis

Tiled confocal stacks were first stitched by TeraStitcher (https://abria.github.io/TeraStitcher/; Bria and Iannello, 2012). The stitched volumes were rotated to match the standard orientation (up: anterior, left: lateral). Using surface vasculature, the field of views of each 2p session from the same mouse were located in the confocal volume. By carefully following the descending blood vessels in both 2p and confocal volumes in the red channel, the imaged depths were reached, and the imaged axon segments were identified in the green channel. From those identified axon segments, the complete axon arbor was manually traced using TeraFly/Vaa3d software (https://alleninstitute.org/what-we-do/brain-science/research/products-tools/vaa3d/).

To quantify the morphological features, we calculated the total length, maximum branching number, 2D diameter, and density for each reconstructed axon arbor. To calculate 2D diameter, we first collapsed all segments into a 2D xy plane. Then, the diameter of a circle centered at the center of mass that encompassed 95% of all segments was defined as the axon’s 2D diameter. The density was defined as the total length divided by the volume of the cylinder constructed by extruding the circle across the cortical depth. We also calculated total length and density at different cortical depth. Specifically, we calculated total length and density with 10 μm step from 0 – 600 μm to generate the depth profile. We also calculated mean density at different depth ranges to compare the layer specificity (superficial layer: 0 – 150 μm; middle layer: 150 – 350 μm; deep layer: 350 – 600 μm).

### QUANTIFICATION AND STATISTICAL ANALYSIS

The imaging preprocessing, nwb packaging, bouton clustering, receptive field analysis, grating response analysis, functional type classification, and axon morphology analysis were performed by a custom-written python package “NeuroAnalysisTools” (https://github.com/zhuangjun1981/NeuroAnalysisTools).

To control the variability across imaging planes (different axon/bouton density, vasculature pattern, expression level), most statistics were extracted from each plane and separated for different functional types if necessary. For statistics that produced only one number from each imaging plane (e.g., bouton count), they were presented as mean ± standard deviation. For statistics that a population distribution can be drawn from each imaging plane (gDSI, gOSI, peak SF/TF, RF strength, bouton per cluster, max bouton distance, axon coverage area), the mean for each plane was calculated first and mean ± s.e.m. was reported across imaging planes. The comparisons between different functional types within an imaging plane were performed by Wilcoxon rank-sum test, and comparisons between imaging planes were performed by Mann-Whitney U test, if not otherwise stated. Nonetheless, we have verified our results of these two tests by paired t test and independent t test respectively, and they all agreed with the non-parametric tests (not shown). The statistical details can be found in the results and figure legends. All p values reported in results and figure legends are two-tailed likelihood of null hypothesis.

## Supplementary Material

1

## Figures and Tables

**KEY RESOURCES TABLE:** 

REAGENT or RESOURCE	SOURCE	IDENTIFIER

Antibodies		

Chicken polyclonal anti-GFP antibody	Abcam	Cat#ab13970; RRID:AB_300798
Alexa Fluor 488 donkey anti-chicken IgG	Jackson Immunoresearch	Cat#703-545-155; RRID:AB_2340375

Bacterial and virus strains		

AAV1 -Syn-FLEX-GCaMP6s	Chen et al., 2013	Addgene: 100845-AAV1
AAV1 -CAG-FLEX-GCaMP6s	Chen et al., 2013	Addgene: 100842-AAV1
AAV9-hSyn-Cre	James M. Wilson Lab	Addgene: 105553-AAV9

Chemicals, peptides, and recombinant proteins		

1 μm Fluorescent beads	Thermo Fisher	F8821
Lectin, DyLight® 649	Vector Laboratories	DL1178
Dextran Texas Red	Thermo Fisher	D3328

Experimental models: Organisms/strains		

Ai14(RCL-tdT)	The Jackson Laboratory	JAX: 007914
Vipr2-IRES2-Cre-neo	This manuscript	We are in the process of depositing this mouse line to Jackson Laboratory. Please contact Dr. Bosiljka Tasic (bosiljkat@alleninstitute.org) for the availability of this mouse line.

Recombinant DNA		

AAV pCAG-mRuby3-WPRE	This manuscript	Addgene: 107744

Software and algorithms		

ScanImage	Vidrio Technologies	http://scanimage.vidriotechnologies.com/display/SIH/ScanImage+Home
retinotopic_mapping	This manuscript	https://doi.org/10.5281/zenodo.5512971
PsychoPy	PsychoPy	https://www.psychopy.org/
Neu roAnalysisTools	This manuscript	https://doi.org/10.5281/zenodo.5512966
CaImAn	GitHub	https://github.com/flatironinstitute/CaImAn
ainwb	GitHub	https://github.com/AllenInstitute/nwb-api
Scipy	Scipy	https://www.scipy.org/
TeraStitcher	GitHub	https://abria.github.io/TeraStitcher/
Vaa3d/TeraFly	Allen Institute	https://alleninstitute.org/what-we-do/brain-science/research/products-tools/vaa3d/
Numpy	Numpy	https://numpy.org/
Fiji software	Fiji	https://flji.sc/Fiji
Python v2.7, v3.8	Python	https://www.python.org

## Highlights

- DS/OS and LS axons form two major functional types of inputs from dLGN to V1
- Both DS/OS and LS boutons are distributed from superficial to middle layers in V1
- Single-axon arbors are reconstructed for DS/OS and LS axons
- DS/OS axons have denser arbors in the middle layers than LS axons
