# Supplementary material for: Laminar distribution and arbor density of two functional classes of thalamic inputs to primary visual cortex
Source: Cell Rep. Author manuscript; Available in PMC 2021 Nov 7. (PMC8572142; doi:10.1016/j.celrep.2021.109826)
Supplement: 1 [file NIHMS1749563-supplement-1.pdf]

Cell Reports, Volume *37*

Supplemental information

Laminar distribution and arbor density of two functional classes of thalamic inputs to primary visual cortex

Jun Zhuang, Yun Wang, Naveen D. Ouellette, Emily E. Turschak, Rylan S. Larsen, Kevin T. Takasaki, Tanya L. Daigle, Bosiljka Tasic, Jack Waters, Hongkui Zeng, and R. Clay Reid

Depth (um)	Imaging Plane	OS/DSnLS	LSnOS/DS	LS&OS/DS	nLSnOS/DS	Total
50	14	502 (0.142)	1999 (0.565)	148 (0.042)	888 (0.251)	3537
100	19	399 (0.178)	1175 (0.525)	81 (0.036)	584 (0.261)	2239
150	14	425 (0.177)	1297 (0.541)	116 (0.048)	561 (0.234)	2399
200	19	487 (0.155)	1779 (0.566)	196 (0.062)	683 (0.217)	3145
250	14	484 (0.167)	1527 (0.527)	212 (0.073)	674 (0.233)	2897
300	18	457 (0.178)	1494 (0.581)	122 (0.047)	497 (0.193)	2570
350	9	470 (0.245)	772 (0.403)	149 (0.078)	525 (0.274)	1916
400	7	345 (0.273)	478 (0.378)	101 (0.080)	340 (0.269)	1264
all	114	3569 (0.179)	10521 (0.527)	1125 (0.056)	4752 (0.238)	19967

Table S1. Related to **Figure 1**. Imaging plane and ROI counts at each depth. Number in parentheses: ratio to the total ROI numbers at given depth.

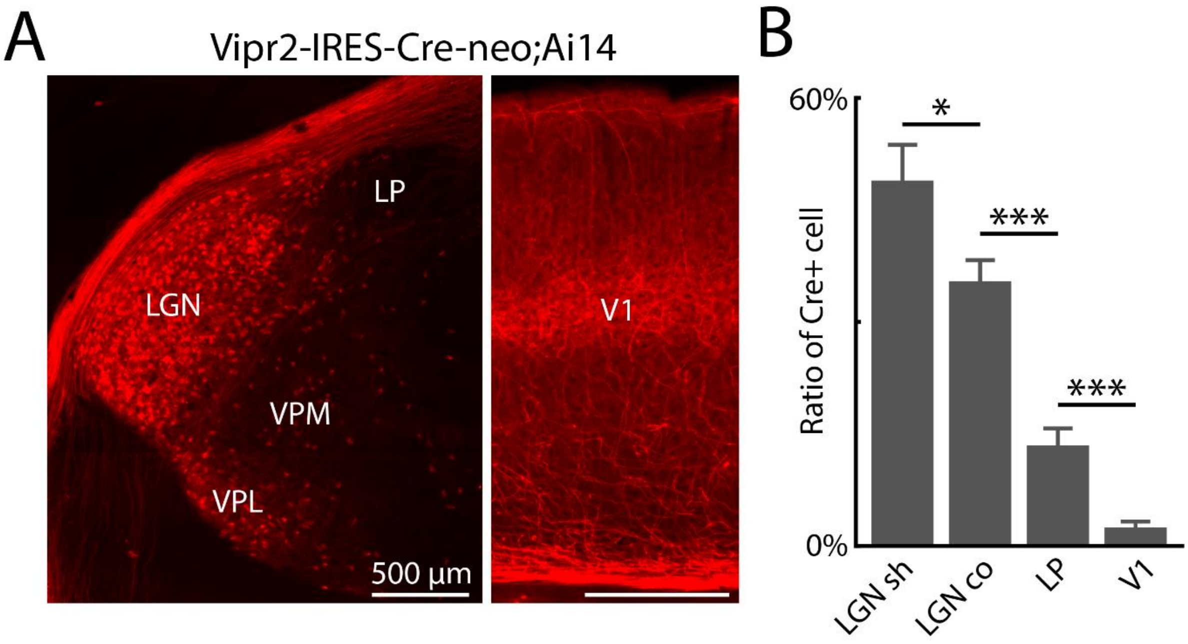

Figure S1. Cre expression characterization of Vipr2-IRES-Cre-neo mouse line. Related to **Figure 1**.

(A) Coronal sections with tdTomato positive cells in thalamus (left) and axons in V1 (right) from a Vipr2-IRES-Cre-neo;Ai14 mouse.

(B) Ratio of Cre+ cells to Nissl labeled cells in visual thalamus and V1. Data from Allen Brain Atlas Transgenic Characterization dataset (experiments: 576523754, 576524006). 10 fields of view were manually selected for each brain region. For each field of view, Cre+ cells and Nissl+ cells were manually counted and the ratio between the two were calculated. dLGN sh: dLGN shell. dLGN co: dLGN core. LP: lateral posterior nucleus of thalamus. V1: primary visual cortex. Bar graph: mean ± s.e.m. Independent t-test: dLGN sh vs. dLGN co: t=2.3, p=0.034; dLGN co vs. LP, t=5.8, p=1.8×10^{-5}; LP vs. V1, t=4.4, p=0.00037.

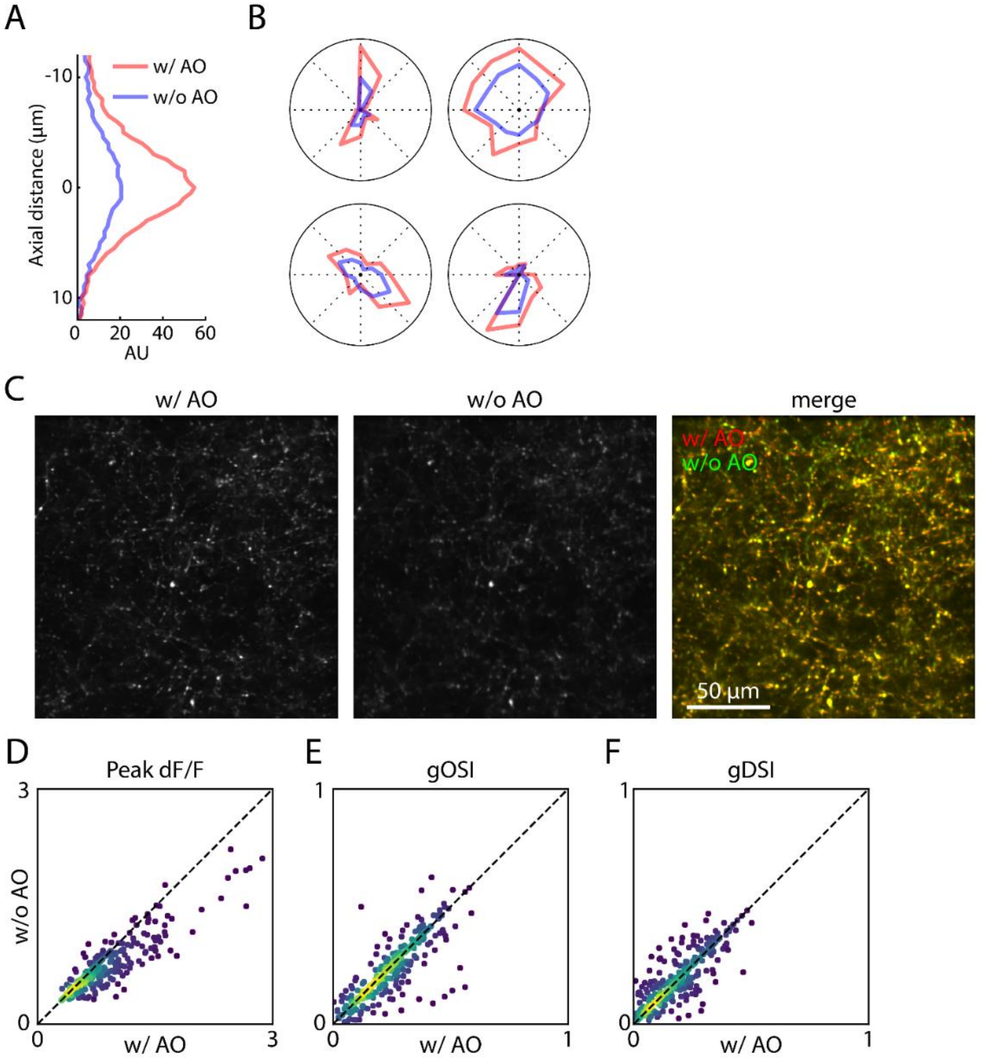

Figure S2. Comparisons of response amplitude, direction selectivity, and orientation selectivity between conditions with and without adaptive optics (AO). Related to **Figure 1**.

(A) Axial profile with and without AO of 1 μm beads deposited between coverglass and brain surface in an awake mouse.

(B) Example direction tuning curves from 4 example boutons under with AO and without AO conditions.

(C) Mean projection of an example imaging plane with and without AO. Although the signal amplitude was higher with AO, the structure patterns were almost identical between images with and without AO, indicating they were at the same cortical depth. In the merged image, lookup tables were adjusted to match the brightness between with AO and without AO conditions.

(D – F) Comparisons of peak dF/F, gOSI, and gDSI, respectively, between with AO and without AO conditions. Two imaging planes in two mice (300 μm and 350 μm below pia). In AO conditions, laser beam wave front was corrected separately for each mouse. With AO vs. without AO, 328 boutons; peak dF/F: 0.54 ± 0.34 vs. 0.46 ± 0.25, t = 10.34, $p = 7x10^{-22}$; gOSI: 0.24 ± 0.12 vs. 0.23 ± 0.12, t = 2.92, p = 0.004; gDSI: 0.18 ± 0.11 vs. 0.18 ± 0.11, t = 0.49, p = 0.432; mean ± standard deviation, paired t-test.

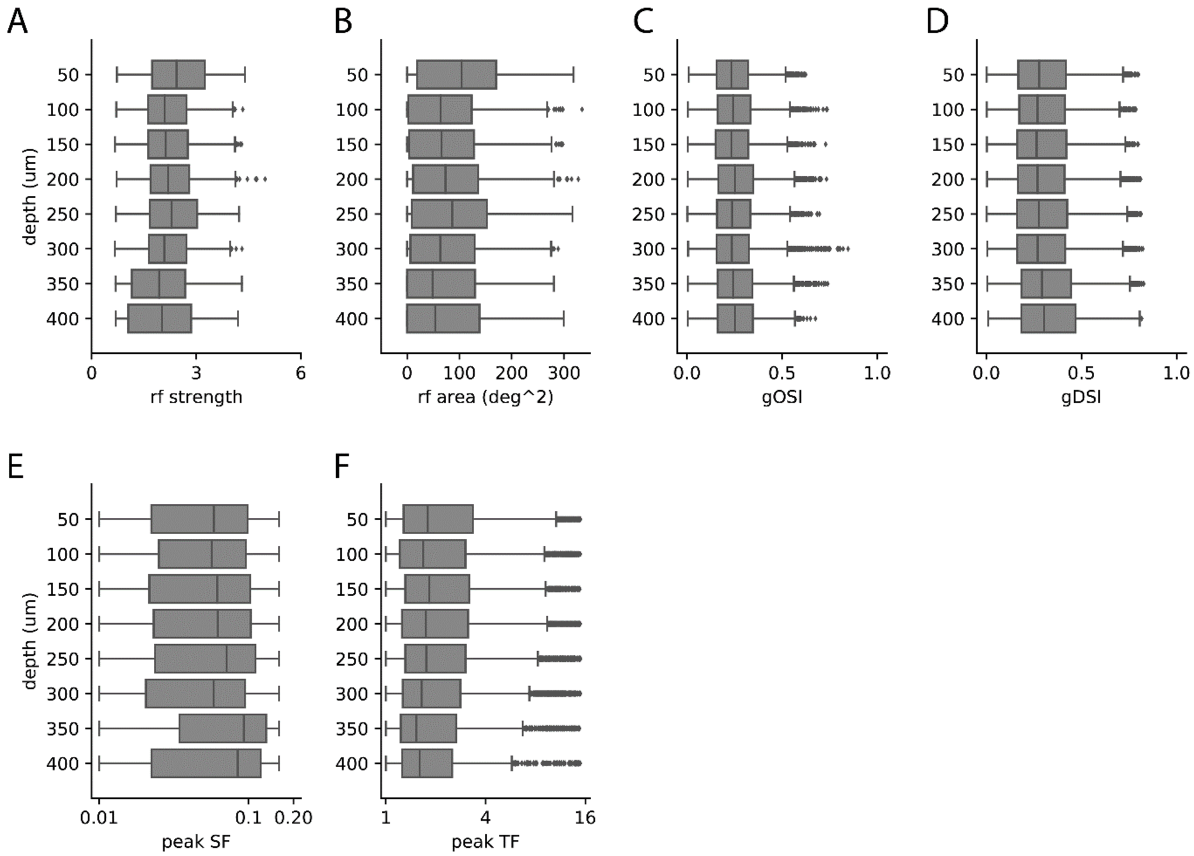

Figure S3. Response properties across depth. Related to **Figure 1.**

(A - F) Response property distributions of individual boutons across cortical depth. middle line: median; box: 2nd and 3rd quartiles; whisker: 1.2 inter-quartile range; dot: outlier. For gDSI, gOSI, peak SF, and peak TF, only boutons with significant responses to drifting gratings (Methods) were included. One-way ANOVA, RF strength: F=1.47, p=0.19; gDSI: F=0.42, p=0.89; gOSI: F=1.76; p=0.10; peak SF: F=3.79, p=0.001; peak TF: F=1.70, p=0.12. See Table S1 for ROI counts.

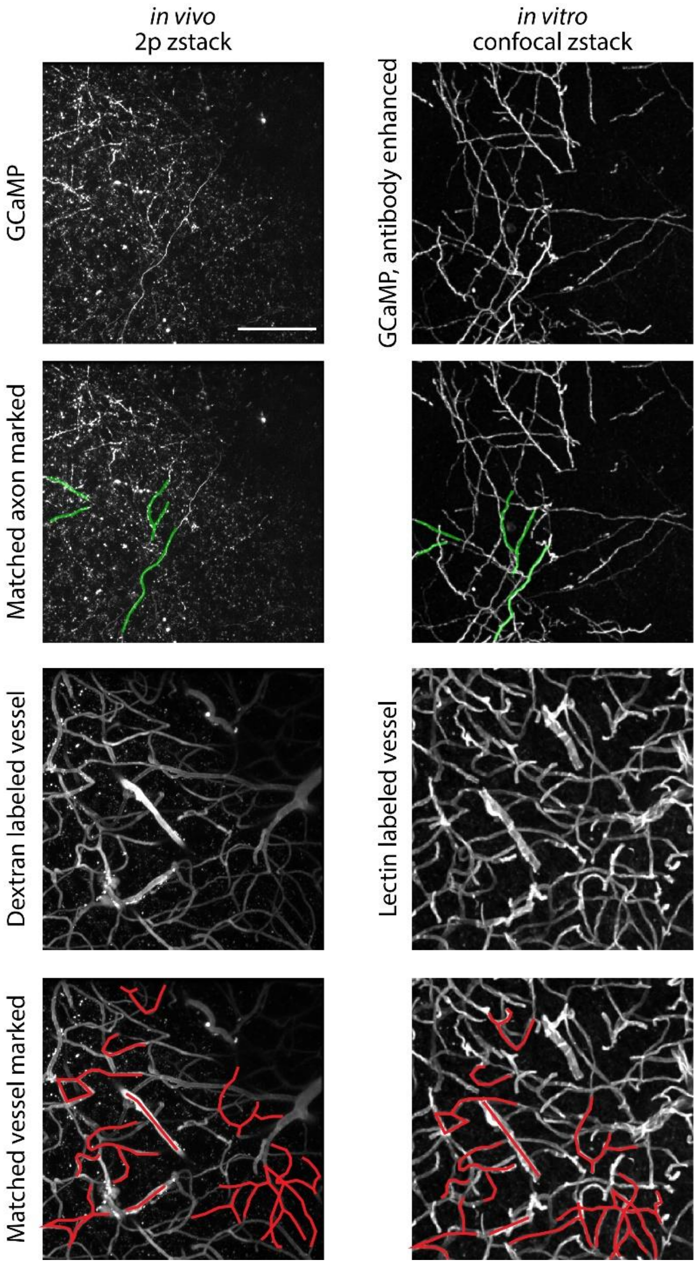

Figure S4. Coregistration between *in vivo* two-photon volumes and *in vitro* confocal volumes. Related to **Figure 3** and **STAR Methods**.

For *in vivo* two-photon imaging (left column), the dLGN axons were sparsely labeled with GCaMP by AAV viral labeling and blood vessels were labeled by fluorescence dye (dextran-Texas Red). For *in vitro* confocal imaging (right column), the imaged tissue volume was fixed and cleared, the GCaMP signal was enhanced by antibody labeling, and blood vessels were labeled by fluorescence dye (lectin-DyLight). Blood vessels labeled in both imaging modalities were used as fiducial structure for locating the same region in both two-photon and confocal images (bottom row, red lines), and the same axons imaged *in vivo* were identified in the confocal volumes (second row, green lines). Scale bar: 50 µm.
